# Incubation of Oxycodone Craving Following Adult-Onset and Adolescent-Onset Oxycodone Self-Administration in Male Rats

**DOI:** 10.3389/fnbeh.2021.697509

**Published:** 2021-06-23

**Authors:** Rachel D. Altshuler, Kristine T. Garcia, Xuan Li

**Affiliations:** Department of Psychology, University of Maryland, College Park, College Park, MD, United States

**Keywords:** oxycodone, craving, self-administration, adolescent, adult rats, housing

## Abstract

Relapse is a major obstacle to curb the ongoing epidemic of prescription opioid abuse. We and others previously demonstrated that oxycodone seeking in adult rats progressively increases after abstinence from oxycodone self-administration (incubation of oxycodone craving). In humans, the onset of oxycodone use in adolescents may increase individuals’ vulnerability to later opioid addiction. However, little is known about incubation of oxycodone craving after adolescent-onset oxycodone self-administration in rats. In the first study, we trained single-housed adolescent (postnatal day 35 at start) and adult (postnatal day 77 at start) male Sprague–Dawley rats to self-administer oxycodone (0.1 mg/kg/infusion, 6 h/day for 10 days) and then tested oxycodone relapse on both abstinence day 1 and day 15. Given that social experience is critical for neurobehavioral development in adolescents, we performed the second study using group-housed adolescent and adult rats. In both studies, we observed no age differences in oxycodone self-administration and incubated oxycodone seeking on abstinence day 15. However, on abstinence day 1, we observed decreased oxycodone seeking in adolescents compared with adults. This pattern of data led to elevated incubation slopes in adolescent rats compared with adult rats. Finally, group-housed rats exhibited attenuated oxycodone seeking compared with single-housed rats on abstinence day 15, but not on day 1. Taken together, these data suggest that adolescents may be resistant to oxycodone relapse during early abstinence, but this resistance dissipates quickly during the transition between adolescent and young adulthood. In addition, group-housing plays a protective role against incubated oxycodone craving.

## Introduction

The opioid epidemic is an ongoing public health crisis, affecting millions of people across the United States. In the past 20 years, over 200,000 overdose-related deaths involved prescription opioids, such as oxycodone (Centers for Disease Control and Prevention [CDC], 2020). One of the main barriers to addressing this opioid epidemic is the prevalence of relapse to drug use (Gostin et al., 2017; Kariisa et al., 2019), often triggered by re-exposure to drug-associated cues (O’Brien et al., 1986). Recently, we and others demonstrated that oxycodone seeking in adult rats progressively increases after abstinence from oxycodone self-administration (Fredriksson et al., 2020, 2021; Altshuler et al., 2021). This incubation of craving phenomenon has also been previously observed in rats with a history of cocaine (Grimm et al., 2001), heroin (Shalev et al., 2001), nicotine (Abdolahi et al., 2010), alcohol (Bienkowski et al., 2004), methamphetamine (Shepard et al., 2004), and sucrose (Grimm et al., 2005) self-administration.

In humans, the onset of drug use, including opioids, often occurs during the adolescent period and young adulthood (Levy, 2019; Miech et al., 2015). Early-onset oxycodone use during adolescence may predispose individuals to vulnerabilities for drug dependence and relapse in the adulthood (Anthony and Petronis, 1995; Clark et al., 1998; Chen et al., 2009). Consistent with this notion, previous work in mice demonstrated that adolescent-onset compared with adult-onset oxycodone self-administration leads to increased oxycodone-induced conditioned place preference (CPP) (Zhang et al., 2016). Chronic non-contingent administration of oxycodone in adolescent mice also increased morphine-induced CPP during adulthood compared with mice receiving oxycodone only in adulthood (Sanchez et al., 2016). In addition, adolescent mice exhibit different sensitivities to the rewarding effects of oxycodone from adult mice (Zhang et al., 2009; Niikura et al., 2013).

However, it is unknown whether adolescent-onset oxycodone self-administration impacts relapse behavior in the adulthood after abstinence. Note that previous studies on the effect of adolescent-onset drug self-administration on drug seeking yielded mixed results (Doherty et al., 2009; Li and Frantz, 2009, 2017; Doherty and Frantz, 2012; Wong and Marinelli, 2016; Madsen et al., 2017; Cho et al., 2020; Guerin et al., 2021) (see details in Discussion), but the majority of the studies do not support the notion that adolescent-onset drug self-administration promotes relapse behavior in adulthood. Here, we aimed to compare incubation of oxycodone craving following adolescent-onset and adult-onset oxycodone self-administration. In the first experiment, we used single-housed rats, which was the standard housing condition for most incubation studies using adult rats (Pickens et al., 2011; Wolf, 2016; Altshuler et al., 2020, 2021; Fredriksson et al., 2020). We found no age differences in incubated oxycodone seeking on abstinence day 15. Next, we followed up on this finding with the second experiment using group-housed rats. This is based on numerous studies demonstrating that social experience (e.g., social play) contributes to neurobehavioral development in adolescent rats (Vanderschuren et al., 2016; Burke et al., 2017) and environmental enrichment involving social housing attenuates drug seeking in adult rats (Thiel et al., 2009, 2010, 2012; Hofford et al., 2014; Imperio et al., 2018; Frankowska et al., 2019; Mastrogiovanni et al., 2021). In particular, previous studies found that the single-housing condition offsets the age difference in incubation of cocaine craving, an effect observed in pair-housed rats (Li and Frantz, 2009, 2017).

## Materials and Methods

### Subjects

Male Sprague–Dawley rats (Charles River) arrived in our animal facility at either postnatal day (P) 21 (total *n* = 62) or 63 (total *n* = 49). Adolescent rats were at P 35 and adult rats were at P 77 at the beginning of the self-administration training. In Experiment 1, we group-housed rats 4 per cage before surgery and then housed them individually after surgery (single-housed condition). In Experiment 2, rats were group-housed four per cage before and after surgery (group-housed condition). In the case that we lost cage mates due to health-related issues, we ensured that there were no less than three rats per cage in the group-housed condition. All rats were maintained under a reverse 12:12-h light/dark cycle with food and water freely available. We performed the experiments under the protocols approved by the University of Maryland College Park Animal Care and Use committee. We excluded rats due to catheter patency (adult = 6, adolescent = 5), poor training (adult = 9, adolescent = 13), and health-related issues (adult = 1, adolescent = 4). The number of rats reported herein refers to rats included in the statistical analysis.

### Intravenous Surgery

We anesthetized the rats with isoflurane gas (5% induction; 2–3% maintenance) and inserted silastic catheters into the rat’s jugular vein, as previously described (Caprioli et al., 2017; Li et al., 2018). We injected the rats with ketoprofen (2.5 mg/kg, s.c.) after surgery to relieve pain and inflammation; we allowed the rats to recover 5–7 days before oxycodone self-administration training. During the recovery and training phases, we flushed the catheters every 24–48 h with gentamicin (Butler Schein; 5 mg/ml) dissolved in sterile saline.

### Apparatus

We trained the rats in self-administration chambers located inside sound-attenuating cabinets and controlled by a Med Associates (Georgia, VT, United States) system. Each chamber has two levers located 8–9 cm above the floor. During self-administration training, presses on the retractable (active) lever activated the infusion pump (which delivered an oxycodone infusion); presses on the stationary (inactive) lever were not reinforced with the drug. For oxycodone intravenous infusions, we connected each rat’s catheter to a liquid swivel (Instech) *via* polyethylene-50 tubing, protected by a metal spring. We then attached the liquid swivel to a 20-ml syringe *via* polyethylene-50 tubing and to a 22-gauge modified needle (Plastics One, VA). To promote lever pressing for adolescents, we set the active lever 3 cm lower during the first 4 days of training.

### Oxycodone Self-Administration Training

We used a training procedure previously described by Altshuler et al. (2021). We trained the rats to self-administer oxycodone for 6 h per day, under a fixed-ratio-1 (FR1) with 20-s timeout reinforcement schedule. Each training session included six 1-h sessions with 10 min off in between sessions. We dissolved oxycodone (kindly provided by National Institute on Drug Abuse Drug Supply Program) in saline (10 mg/ml), and the rats self-administered oxycodone at a dose of 0.1 mg/kg/infusion over 3.5 s (0.10 ml/infusion). We trained the rats for 10 sessions over an 11-day period (off day between the 5th and 6th day). We used Brevital (3 to 4 mg/kg) to check catheter patency for low responders during the training. We only included rats with an average number of infusions above 20 across 10 training days and across the last 3 training days for the following behavioral procedures.

The daily training sessions started at the onset of the dark cycle and began with the extension of the active lever and the illumination of the red house light. The house light remained on for the duration of the 6-h session. During training, active lever presses led to the delivery of an oxycodone infusion and a compound 5-s tone-light cue (the tone and light modules were located above the active lever). During the 20-s timeout, we recorded the non-reinforced lever presses. We set 90 infusions as the maximum for each 6-h session to prevent overdose. The red house light was turned off and the active lever retracted after the rats received the maximum infusions or at the end of the 6-h session.

### Abstinence Phase

During the abstinence phase, we housed the rats either individually or in groups in the animal facility and handled them two to three times per week.

### Relapse Test

We conducted all relapse tests immediately after the onset of the dark cycle. The sessions began with the extension of the active lever and the illumination of the red house light, which remained on for the duration of the session. Active lever presses during testing (the operational measure of drug seeking in incubation of craving studies) resulted in contingent presentations of the tone-light cue, previously paired with oxycodone infusions, but did not result in drug infusions.

### Experimental Procedures

**FIGURE 1:**
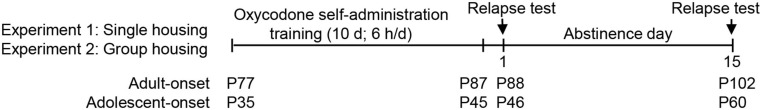
Experimental timeline. We trained adult (P77) and adolescent (P35) rats to self-administer oxycodone for 10 days and tested them for relapse on abstinence days 1 and 15 under either single-housing condition (Experiment 1) or group-housing condition (Experiment 2).

All rats first received intravenous surgeries. Next, we trained them to self-administer oxycodone for 10 days. On abstinence day 1, we tested the rats for relapse in a 30-min session. On abstinence day 15, we tested the rats for relapse in a 60-min session. Note that we used a 30-min session on abstinence day 1 to minimize the effect of extinction learning that could decrease drug seeking on abstinence day 15 (Li et al., 2018; Fredriksson et al., 2020). We performed Experiment 1 (single-housed condition) in four independent runs (total adult = 21, total adolescent = 26). We performed Experiment 2 (group-housed condition) in three independent runs (total adult = 12, total adolescent = 14). The detailed experimental timeline is shown in Figure 1.

### Statistical Analysis

We analyzed the data with SPSS (version 24) mixed ANOVAs or *t*-test as appropriate. We followed significant interaction or main effects with Fisher PLSD tests. For the repeated measures analyses of the training data, we replaced 24 outlier values of inactive lever presses with the group mean for a given training day. We defined outliers as three median absolute deviations (MADs) above the group median (Leys et al., 2013), and we only replaced one outlier (the highest value above the threshold) for each training day. For rats disconnected from swivels on training day 8 or 9, we extended their training for an additional 1 to 2 days. We replaced their day 10 training data with the mean of the last two or three training days. For lever presses during relapse tests and incubation slopes, we also excluded one outlier above and/or below the threshold (defined by above or below 3 MADs from the group median). In addition, we used the data from the day 1 relapse test and the first 30 min of the day 15 relapse test to calculate incubation slope. The equation is “Incubation slope = (active lever presses on abstinence day 15 − active lever presses on abstinence day 1)/(15 − 1),” Finally, we indicate the between- and within-subject factors of the different analyses in the Results section. All statistical comparisons are listed in Supplementary Table 1.

## Results

### Incubation of Oxycodone Craving Following Adult-Onset and Adolescent-Onset Oxycodone Self-Administration in Single-Housed Rats

The goal of Experiment 1 was to examine whether adolescent-onset oxycodone self-administration leads to changes in oxycodone seeking after abstinence compared with adult-onset oxycodone self-administration under the single-housing condition. To achieve this goal, we used a within-subject design and tested rats for oxycodone seeking (relapse tests) on abstinence day 1 and 15 (Figure 1).

#### Oxycodone Self-Administration (Figures 2A,B)

**FIGURE 2:**
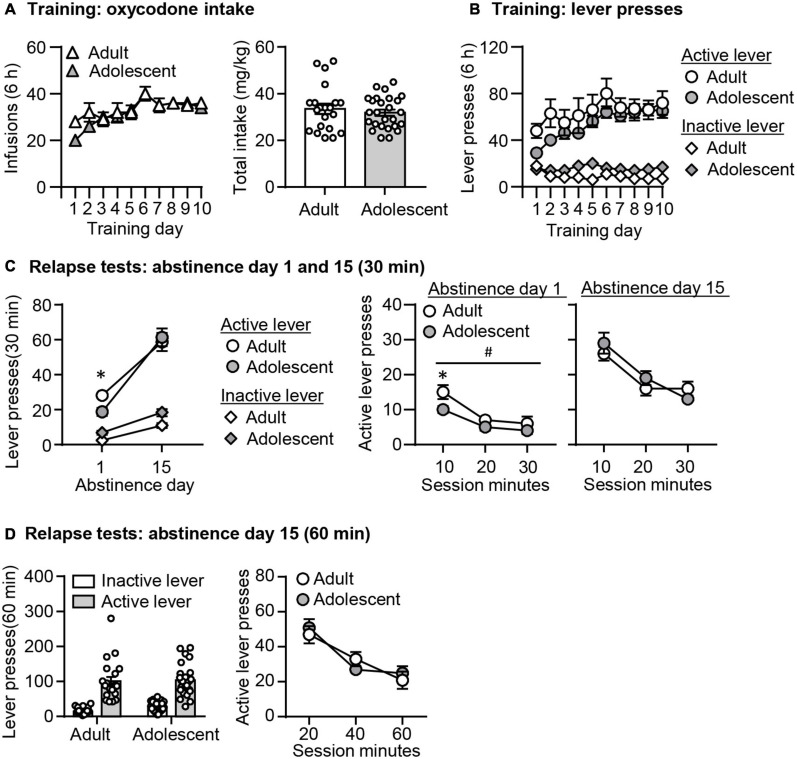
Incubation of oxycodone craving following adult-onset and adolescent-onset oxycodone self-administration under the single-housing condition. **(A,B)** Data are mean ± SEM number of oxycodone (0.1 mg/kg/infusion) infusions, total oxycodone intake, and lever presses during the ten 6-h daily self-administration sessions for Exp. 1. During training, active lever presses were reinforced on an FR1 20-s timeout reinforcement schedule, and oxycodone infusions were paired with a 5-s tone-light cue. **(C)** Relapse test on abstinence days 1 and 15: Data are mean ± SEM of active and inactive lever presses during the 30-min relapse test on abstinence day 1 and the first 30 min of the 60-min relapse test on abstinence day 15. ^#^Main effect of Age; *Different from Adult, *p* < 0.05. **(D)** Relapse test on abstinence day 15: Data are mean ± SEM of active and inactive lever presses during the 60-min relapse test. During testing, active lever presses led to contingent presentations of the tone-light cue previously paired with oxycodone infusions during training, but not oxycodone. *n* = 19–25/group.

Both adult-onset and adolescent-onset rats increased their oxycodone intakes similarly across training days. We analyzed the infusion data with the between-subject factor of Age (adult, adolescent) and within-subject factor of Training day (1–10). We observed a significant effect of Training day (*F*_9,405_ = 12.378, *p* < 0.001), but no significant effect of Age or interaction between these two factors (*p* > 0.05, Figure 2A). We also found no age differences in total oxycodone intake during training (*p* > 0.05, Figure 2A). In addition, active lever presses but not inactive lever presses in both groups increased during training. The analysis of the lever press included the between-subject factor of Age (adult, adolescent) and within-subject factors of Training day (1–10) and Lever (active lever, inactive lever). We observed a significant interaction between Lever and Training Day (*F*_9,405_ = 8.866, *p* < 0.001), but no effects of Age or interaction among these three factors (*p* > 0.05, Figure 2B).

#### Relapse Tests (Figures 2C,D)

In both groups, oxycodone seeking was significantly higher on abstinence day 15 than on day 1. However, oxycodone seeking in the adolescent group was lower than the adult group on abstinence day 1, while no age differences in oxycodone seeking were observed on abstinence day 15. As rats were tested for relapse in a 30-min session on abstinence day 1 and a 60-min session on abstinence day 15, we analyzed the data in the following two ways. First, we performed the analysis using the data from the day 1 relapse test, and the first 30 min of the day 15 relapse test. This analysis included the between-subject factor of Age (adult and adolescent), and the within-subject factor of Abstinence day (1 and 15) and Lever (active lever and inactive lever). First, we found no interaction among these three factors (*p* > 0.05, Figure 2C). However, we observed a significant interaction between Lever and Day (*F*_1,39_ = 55.386, *p* < 0.001). *Post hoc* analysis of active lever presses revealed that oxycodone seeking was significantly higher on abstinence day 15 than day 1 in both age groups (adults: *t*_23_ = −8.155, *p* < 0.001; adolescents: *t*_18_ = −6.417, *p* < 0.001). We also observed a significant interaction between Day and Age (*F*_1,39_ = 6.569, *p* = 0.014). *Post hoc* analysis of active lever presses revealed that on abstinence day 1, but not day 15, there was a significant decrease in adolescent rats, compared with adult rats (*t*_42_ = 2.574, *p* = 0.014). In addition, the analysis of the time course data on the active lever included the between-subject factor of Age (adult and adolescent) and the within-subject factor of Session Minutes (10-min intervals). We observed a significant effect of Session Minutes (*F*_2,84_ = 20.002, *p* < 0.001) and Age (*F*_1,42_ = 6.032, *p* = 0.018), but no interaction between these two factors on abstinence day 1 (*p* > 0.05, Figure 2C). On abstinence day 15, there was a significant effect of Session Minutes (*F*_2,86_ = 33.194, *p* < 0.001), but no effect of Age or interaction between these two factors.

In a second analysis, we used the data from the 60-min relapse test on abstinence day 15. The analysis included the between-subject factor of Age (adult and adolescent), and the within-subject factor of Session Minutes (10-min intervals) and Lever (active lever and inactive lever). We found main effects of Session Minutes (*F*_2,90_ = 33.566, *p* < 0.001), but no significant effects of Age or interaction between these two factors (*p* > 0.05, Figure 2D).

In summary, the data from the single-housed condition demonstrated no age differences in adult-onset and adolescent-onset oxycodone self-administration training. In addition, adolescent rats exhibited decreased oxycodone seeking on abstinence day 1 compared with adult rats, but no difference in incubated oxycodone seeking on abstinence day 15.

### Incubation of Oxycodone Craving Following Adult-Onset and Adolescent-Onset Oxycodone Self-Administration in Group-Housed Rats

Based on a recent study suggesting that housing condition contributes to the age differences in incubated cocaine craving (Li and Frantz, 2017), we performed Experiment 2 using the same design as Experiment 1, but under the group-housing condition.

#### Oxycodone Self-Administration (Figures 3A,B)

**FIGURE 3:**
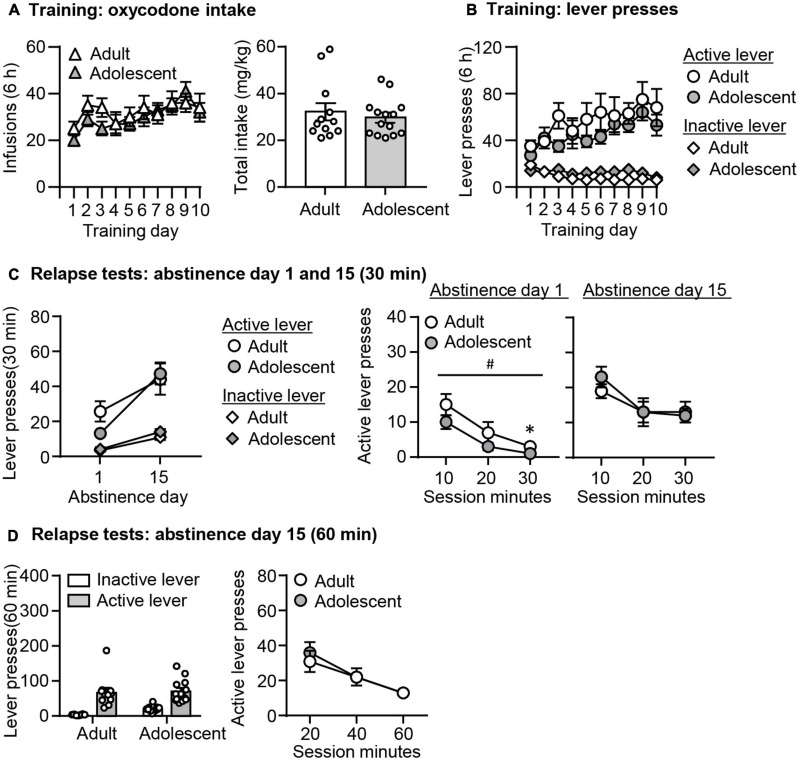
Incubation of oxycodone craving following adult-onset and adolescent-onset oxycodone self-administration under the group-housing condition. **(A,B)** Data are mean ± SEM number of oxycodone (0.1 mg/kg/infusion) infusions, total oxycodone intake, and lever presses during the ten 6-h daily self-administration sessions for Exp. 2. During training, active lever presses were reinforced on an FR1 20-s timeout reinforcement schedule, and oxycodone infusions were paired with a 5-s tone-light cue. **(C)** Relapse test on abstinence days 1 and 15: Data are mean ± SEM of active and inactive lever presses during the 30-min relapse test on abstinence day 1 and the first 30 min of the 60-min relapse test on abstinence day 15. ^#^Main effect of Age; *Different from Adult, *p* < 0.05. **(D)** Relapse test on abstinence day 15: Data are mean ± SEM of active and inactive lever presses during the 60-min relapse test. During testing, active lever presses led to contingent presentations of the tone-light cue previously paired with oxycodone infusions during training, but not oxycodone. *n* = 11–14/group.

Both adult-onset and adolescent-onset rats increased their oxycodone intakes similarly across training days. The data analysis is the same as described in Experiment 1. We observed a significant effect of Training day (*F*_9,216_ = 5.492, *p* = 0.001), but no significant effect of Age or interaction between these two factors (*p* > 0.05, Figure 3A). We also found no age differences in total oxycodone intake during training (*p* > 0.05, Figure 3A). In addition, active lever presses, but not inactive lever presses in both groups increase during training (Lever × Training day: *F*_9,216_ = 6.652, *p* = 0.001), but no effects of Age or interaction among these three factors (*p* > 0.05, Figure 3B).

#### Relapse Tests (Figures 3C,D)

In both groups, oxycodone seeking was significantly higher on abstinence day 15 than on day 1. Similar to findings in the single-housed condition, oxycodone seeking differed on abstinence day 1 with adolescents being lower than adults, but incubated oxycodone seeking on abstinence 15 was similar across age groups. The data analysis is the same as described in Experiment 1. We found no significant triple interactions among three factors (*p* > 0.05, Figure 3C), but observed a significant interaction between Lever and Day (F_1,24_ = 9.448, *p* = 0.005). *Post hoc* analysis of active lever presses revealed that oxycodone seeking was significantly higher on abstinence day 15 than on day 1 in both age groups (adults; *t*_24_ = −2.320, *p* = 0.041, adolescents: *t*_23_ = −8.155, *p* < 0.001). Furthermore, while the analysis of total lever presses revealed no interactions between Day and Age or a significant main effect of Age (*p* > 0.05, Figure 3C), the analysis of the time course data on the active lever on abstinence day 1 showed a main effect of Age (*F*_1,23_ = 4.744, *p* = 0.04) and Session Minutes (*F*_2,46_ = 35.356, *p* < 0.001), with no interaction between these two factors (*p* > 0.05, Figure 3C). On abstinence day 15, there was a significant effect of Session Minutes (*F*_2,44_ = 11.52, *p* < 0.001), but no effect of Age or interaction between these two factors. Finally, the analysis of the 60-min relapse test on abstinence day 15 showed main effects of Session Minutes (*F*_2,48_ = 23.29, *p* < 0.001), but no significant effects of Age or interaction between these two factors (*p* > 0.05, Figure 3D).

In summary, the data from the group-housed condition are overall similar to what we observed from the single-housed condition. Oxycodone self-administration training was similar between age groups in the group-housed condition. There were no age differences in incubated oxycodone seeking, but lower oxycodone seeking in adolescents than adults on abstinence day 1.

### Comparison of the Development of Incubation of Oxycodone Craving Following Adult-Onset and Adolescent-Onset Oxycodone Self-Administration

**FIGURE 4:**
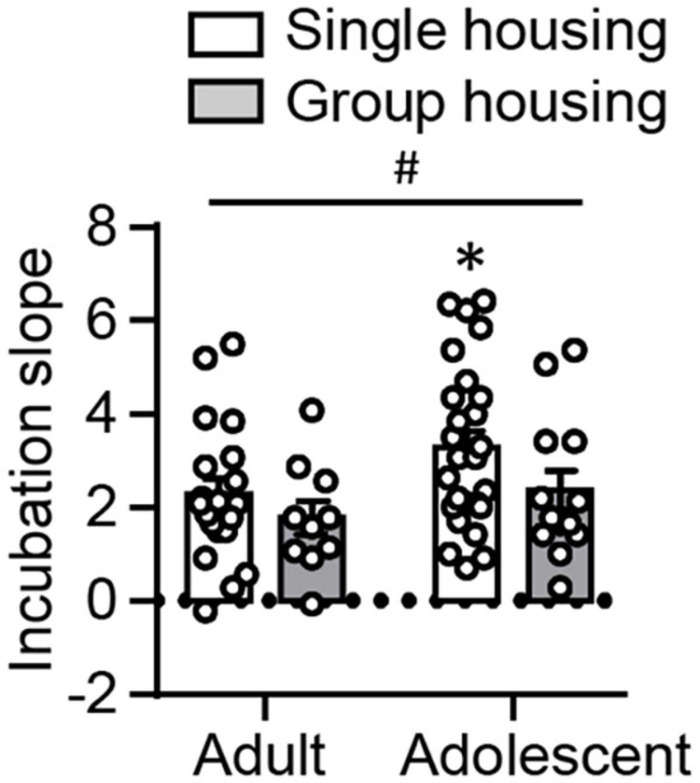
Comparison of incubation slopes following adult-onset and adolescent-onset oxycodone self-administration. Data are mean ± SEM of calculated incubation slopes using the data from the day 1 relapse test, and the first 30 min of the day 15 relapse test. Incubation slope = (active lever presses on abstinence day 15 − active lever presses on abstinence day 1)/(15 − 1). ^#^Main effect of Age; *Different from Adult, *p* < 0.05. *n* = 11–25/group.

In both experiments described above, we observed lower non-incubated oxycodone seeking in adolescents than adults, but no differences in incubated oxycodone seeking. Based on this observation, we are interested in comparing the development of incubation, indexed by incubation slope, following adult-onset and adolescent-onset oxycodone self-administration. We analyzed the incubation slope using the between-subject factors of Age and Housing. We found no interaction between these two factors, but a significant main effect of Age (*F*_1,65_ = 4.084, *p* = 0.047, Figure 4). *Post hoc* analysis showed that under the single-housing condition, adolescent rats exhibited significantly elevated incubation slope compared with adults (*t*_44_ = −2.097, *p* = 0.042), while no statistical significance was detected under group-housing condition (*p* > 0.05). In addition, there was a trend toward significant main effect of Housing (*F*_1,65_ = 3.090, *p* = 0.083). These results suggest that single-housed adolescent rats may have an accelerated incubation compared with single-housed adult rats, which may be interfered under the group-housing condition.

### Effect of Housing Condition on Oxycodone Seeking Following Adult-Onset and Adolescent-Onset Oxycodone Self-Administration

**FIGURE 5:**
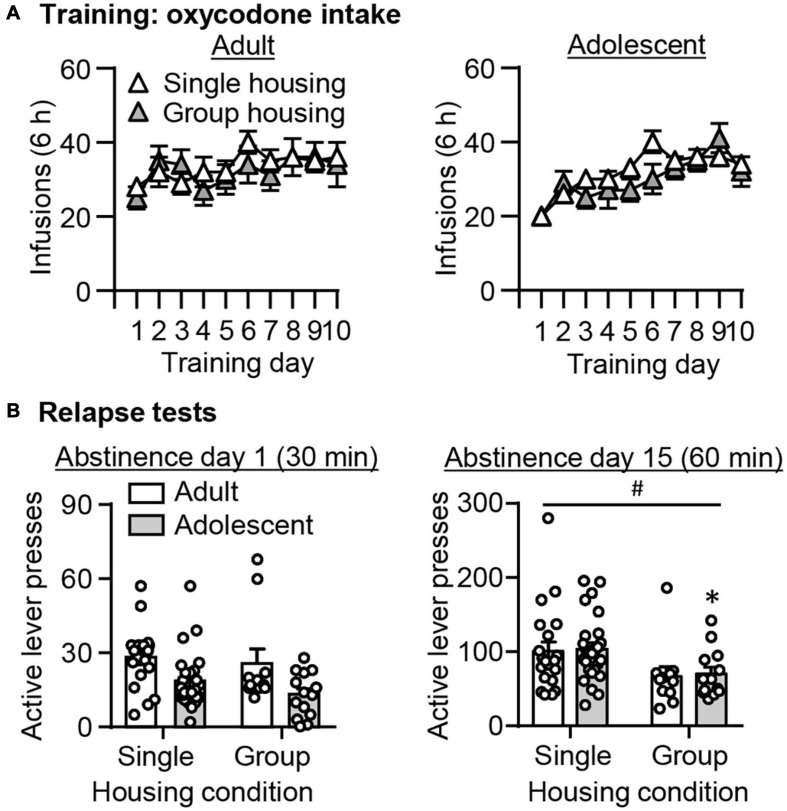
Effect of housing condition on adult-onset and adolescent-onset oxycodone self-administration, and incubation of oxycodone craving. **(A)** Data are mean ± SEM number of oxycodone (0.1 mg/kg/infusion) infusions during the ten 6-h daily self-administration sessions for Adult (left) and Adolescent (right). **(B)** Data are mean ± SEM of active and inactive lever presses during the 30-min relapse test on abstinence day 1 (left) and the 60-min relapse test on abstinence day 15 (right). ^#^Main effect of Housing; *Different from single-housing, *p* < 0.05. *n* = 11–25/group.

Based on substantial evidence demonstrating that environmental enrichment such as social housing attenuates drug seeking across different drug classes (Thiel et al., 2009, 2010; Hofford et al., 2014; Imperio et al., 2018; Frankowska et al., 2019; Westenbroek et al., 2019; Cho et al., 2020; Grimm and Sauter, 2020; Mastrogiovanni et al., 2021), we were interested in examining whether this is also the case in our study. Note that different from most studies in which housing condition is manipulated only during abstinence periods, here the manipulation of housing condition started after intravenous surgeries. First, we observed no effect of housing conditions on oxycodone self-administration across training days (Figure 5A, *p* > 0.05). We analyzed the active lever data from the 30-min relapse test on abstinence day 1, or from the 60-min relapse test on abstinence day 15, using the between-subject factors of Housing condition (single-housing and group-housing) and Age (adult and adolescent). On abstinence day 1, we observed main effects of Age (*F*_1,65_ = 11.439, *p* = 0.001), but no effects of Housing condition or interaction between these two factors (*p* > 0.05, Figure 5B). On abstinence day 15, we observed main effects of Housing condition (*F*_1,67_ = 8.243, *p* = 0.005), but no effects of Age or interaction between these two factors (*p* > 0.05, Figure 5B). *Post hoc* analysis revealed that in adolescent rats, the group-housing condition significantly attenuated oxycodone seeking compared with the single-housing condition (*t*_37_ = 2.381, *p* = 0.023), while there was a trend toward a decrease in oxycodone seeking in group-housed compared with single-housed adult rats (*t*_45_ = 1.745, *p* = 0.090).

Taken together, these analyses demonstrated that group-housing condition attenuated oxycodone seeking on abstinence day 15, but not on day 1. This attenuating effect of group-housing on oxycodone seeking is more robust in adolescents than adults.

## Discussion

In this study, we compared incubation of oxycodone craving following adult-onset and adolescent-onset oxycodone self-administration in single-housed and group-housed rats. Our main finding is that there were no age differences in incubated oxycodone seeking on abstinence day 15, but decreased oxycodone seeking in adolescents compared with adults on abstinence day 1, in both single-housed and group-housed rats. This pattern of data led to the finding that under the single-housing condition, adolescent rats exhibited elevated incubation slopes compared with adult rats. Regarding the effect of housing conditions, we found that on abstinence day 1, the group-housing condition had no effect on oxycodone seeking in both age groups. In contrast, on abstinence day 15, the group-housing condition in adolescent rats significantly attenuated oxycodone seeking compared with the single-housing condition, while in adult rats, the suppressing effect of group-housing was modest. Finally, we observed no age or housing-condition differences in oxycodone self-administration. Overall, these data demonstrate that adolescent rats may be resistant to relapse during early abstinence, but this resistance dissipates quickly once rats reach young adulthood. Additionally, the effect of group-housing was selective for oxycodone seeking after prolonged abstinence, but not during early abstinence.

### Incubation of Drug Craving Following Adolescent-Onset Drug Self-Administration

In both studies, we found no age differences in oxycodone self-administration in rats at a dose of 0.1 mg/kg under extended access condition (6 h/day). We chose this experimental condition based on previous studies from us and others detecting reliable oxycodone self-administration and incubation of oxycodone craving in adult rats (Fredriksson et al., 2020, 2021; Altshuler et al., 2021). In contrast, an early dose-response study showed that adolescent mice self-administered less oxycodone than adult mice across various doses (Zhang et al., 2009). However, direct comparison should be made with caution because of differences in species and experimental procedures between two studies. Regarding the latter, a morphine study previously showed that while adolescent rats self-administer less morphine under the short-access condition (1 h/day), there are no age differences in morphine self-administration under the extended-access condition (Doherty et al., 2009), which is consistent with our finding here. In addition, conflicting results have also been observed under the same heroin self-administration procedures in adolescent rats (Doherty and Frantz, 2012; Doherty et al., 2013), suggesting that the variabilities in reinforcing effects of opioids exist across cohorts in adolescent rats.

Under single-housing condition, we found no age differences in incubated oxycodone seeking on abstinence day 15, which is in line with the previous finding showing no age differences in incubation of cocaine craving following adult-onset and adolescent-onset cocaine self-administration in rats (Madsen et al., 2017). In contrast, previous work from the Frantz group demonstrated that adolescent-onset, compared with adult-onset, drug self-administration leads to attenuated incubation of cocaine and heroin craving in rats (Li and Frantz, 2009; Doherty and Frantz, 2012; Doherty et al., 2013). Three factors could potentially contribute to these discrepancies. The first is the self-administration procedure. While the Frantz group used the limited access drug self-administration procedure (2–3 h/day) (Li and Frantz, 2009; Doherty and Frantz, 2012; Doherty et al., 2013), we and Madsen et al. (2017) used the extended-access procedure (6 h/day), which produces more robust incubation than limited access self-administration (Lu et al., 2004). The second is the housing condition throughout the experiment. Recent work from the Frantz group showed that single-housing condition, used here and also by Madsen et al. (2017), could offset the age differences in incubated cocaine seeking previously observed under group-housing condition (Li and Frantz, 2017). To explore this possibility, we examined incubation of oxycodone craving under group-housing condition, similar to the experimental condition used by Li and Frantz (2009). Again, we found no age differences in incubated oxycodone seeking on abstinence day 15, which further validates our finding under single-housing condition.

The third potential factor is the presence of cues during relapse tests. Doherty and Frantz (2012) found that while adolescent-onset heroin self-administration leads to attenuation of incubated heroin seeking without the presence of cues, there are no age differences in extinction responding in the presence of cues, which is in line with our findings. Stimuli-specific age differences are also observed previously in cocaine studies. For example, adolescent-onset cocaine self-administration leads to decreased cue-induced reinstatement (Li and Frantz, 2009, 2017; Anker and Carroll, 2010), increased stress-induced reinstatement (Anker and Carroll, 2010; Wong and Marinelli, 2016), and no change in context-induced reinstatement of cocaine seeking (Cho et al., 2020) in rats. Taken together, these results suggest that instead of enhancing vulnerability for all types of future relapse, adolescent-onset drug exposure might bidirectionally alter the individuals’ sensitivity to certain relapse-eliciting stimuli in a drug-specific manner.

An unexpected finding here is that while no age differences were observed regarding total oxycodone intake, adolescent rats exhibited attenuated oxycodone seeking on abstinence day 1, compared with adult rats. This result suggests that adolescents might be resistant to oxycodone seeking during early abstinence after oxycodone self-administration, which contrasts with no age differences in oxycodone seeking observed on abstinence day 15. A key difference between these two time points is that on abstinence day 1, the adolescent group was still in adolescent period, but they reached young adulthood on abstinence day 15 (Spear, 2000; McCutcheon and Marinelli, 2009). Therefore, we speculate that this resistance to oxycodone seeking may be due to behavioral characteristics in the adolescent period, such as enhanced exploratory behavior, novelty-seeking, impulsivity, and cognitive and behavioral flexibility (Douglas et al., 2003; Doremus-Fitzwater et al., 2012; Simon et al., 2013; Westbrook et al., 2018). Consistent with our finding, several studies have shown that adolescent rats, compared with adults, are resistant to forming reward-seeking habits (Anderson and Spear, 2011; Doremus-Fitzwater and Spear, 2011; Naneix et al., 2012; Serlin and Torregrossa, 2015; Rode et al., 2019). This suppressed drug-seeking behavior during early abstinence in adolescent rats might also be specific to oxycodone, because previous cocaine and heroin studies all found no differences in drug seeking on abstinence day 1 (Li and Frantz, 2009; Doherty and Frantz, 2012; Doherty et al., 2013; Madsen et al., 2017).

Finally, taking data from abstinence days 1 and 15 together, we identified an elevated incubation slope in adolescent-onset rats, compared with adult-onset rats, suggesting that adolescent-onset rats accelerated the development of incubation during abstinence. Therefore, one possibility is that adolescent-onset rats might exhibit enhanced oxycodone seeking than adult-onset rats in relapse tests beyond abstinence day 15. Although we cannot exclude this possibility, we believe this is unlikely because incubated oxycodone seeking in adult rats plateaus on abstinence day 15, without future increase on abstinence day 30 (Fredriksson et al., 2020). Alternatively, we speculate that this acceleration only occurs between abstinence days 1 and 15, during which adolescent rats transition into young adulthood and start exhibiting adult-like behaviors. It is of note that *post hoc* analysis identified the significant differences of the incubation slope between ages only under the single-housing condition, but not under the group-housing condition. Together with the trending significant main effect of Housing (*p* = 0.083), it is possible that the suppressing effect of the housing condition on oxycodone seeking on abstinence day 15 (see below) interferes with the development of incubation.

### Role of Group-Housing on Drug Seeking

First, we observed no main effect of housing conditions on oxycodone self-administration, which is in line with a recent study showing no effect of environmental enrichment on heroin self-administration in adult rats (Imperio et al., 2018). In contrast, an early study showed that group-housing decreases acquisition of heroin self-administration in adult rats (Bozarth et al., 1989). This discrepancy may be due to different drug doses and number of rats in the group-housing condition across these studies. It is noted that substantial literatures have demonstrated that rats reared in enriched environment show decreased self-administration to cocaine (Green et al., 2010), methamphetamine (Westenbroek et al., 2019), amphetamine (Bardo et al., 2001), methylphenidate (Alvers et al., 2012), and remifentanil (Hofford et al., 2017). However, in these studies, self-administration behavior was assessed after several weeks of environmental enrichment when rats reached young adulthood, instead of being assessed concurrently during development. Therefore, our data suggest that reinforcing effect of oxycodone may overwrite the protective effect of social housing against drug intake during development.

On abstinence day 15, we found that group-housing attenuated oxycodone seeking. Several studies previously demonstrated that environmental enrichment, using a combination of group-housing and enrichment apparatuses in home cages, decreases cue-induced seeking to cocaine (Thiel et al., 2009, 2010, 2012), methamphetamine (Hofford et al., 2014), and sucrose (Grimm and Sauter, 2020), and heroin-priming induced heroin seeking (Imperio et al., 2018), compared with single-housing conditions in rats. Recent studies also showed group-housing alone in rats decreases cue-induced cocaine (Frankowska et al., 2019), methamphetamine (Westenbroek et al., 2019), nicotine, and sucrose (Mastrogiovanni et al., 2021) seeking, and context-induced cocaine seeking (Cho et al., 2020). Our result here is in line with these previous findings. It is of note that *post hoc* analysis identified the significant differences of oxycodone seeking on abstinence day 15 between housing conditions only in adolescent rats, with a trend toward statistical significance in adult rats (*p* = 0.090). The modest effect in adult rats may be due to the timing of housing manipulation, as discussed in detail below.

On abstinence day 1, we found no main effect of housing condition. The lack of statistical significance in adolescent rats could be due to a floor effect attributed by low lever responding on abstinence day 1. In adult rats, our finding is inconsistent with previous studies showing that environmental enrichment with a combination of social housing and enrichment apparatuses in home cages decreases cue-induced cocaine seeking (Chauvet et al., 2012) and sucrose seeking (Grimm and Sauter, 2020) on abstinence day 1, respectively. Beside the possible contribution from the enrichment apparatuses, another main factor that contributes to this discrepancy could be the timing of housing manipulations. In our study, housing conditions were maintained throughout the study, to examine whether social housing differentially affects incubation of oxycodone craving in adolescents and adults. However, Chauvet et al. (2012) and Grimm and Sauter (2020) employed an intervention approach in which environmental enrichment was implemented during abstinence after self-administration under single-housing conditions. Consistently, an early study showed that the protective effect of environmental enrichment against sucrose seeking after abstinence in adult rats is robust with the latter approach, but absent when housing conditions were maintained throughout the study (Grimm et al., 2008). Taken together, our data suggest that group-housing throughout oxycodone self-administration might protect against oxycodone seeking in adolescents during early abstinence, but not adults. This is possibly because compared with adults, social interaction and play is more rewarding and essential to development in adolescent rats (Panksepp, 1981; Douglas et al., 2004; Vanderschuren et al., 2016; Burke et al., 2017).

## Conclusion

The main finding in our study is that in male rats, adolescent-onset oxycodone self-administration, compared with adult-onset, led to decreased oxycodone seeking during early abstinence, but oxycodone seeking was similar after prolonged abstinence. This result further invalidates the notion that adolescent drug exposure enhances vulnerability to relapse in adulthood, which is in contrast with the enhanced rewarding effect of opioids observed in previous studies (Sanchez et al., 2016; Zhang et al., 2016). Instead, adolescents may be resistant to oxycodone relapse, but this resistance dissipates quickly once they reach young adulthood. In addition, group-housing condition attenuates oxycodone seeking after prolonged abstinence, which extends previous work on the role of environmental enrichment on incubation of drug and sucrose craving (Thiel et al., 2009; Chauvet et al., 2012; Grimm and Sauter, 2020).

One question for future studies is to assess the effect of age and housing on the severity of oxycodone withdrawal during abstinence. Regarding the age, previous studies primarily focusing on morphine and heroin suggest that exposure to opioid during adolescent leads to decreased opioid withdrawal severity, which could be reversed by re-exposure during adulthood (Windisch and Kreek, 2020). In contrast, the social influence on opioid withdrawal severity is more complex and may depend on behavioral procedure (Adler et al., 1975; Coudereau et al., 1997; Broseta et al., 2005; Balter and Dykstra, 2012) or the drug history of the social peers (Windisch and Kreek, 2020). Another question for future studies is that whether the effect observed in our study also generalize to female rats. It is of note that in adult rats, incubation of oxycodone craving is similar between males and females at the behavioral level (Fredriksson et al., 2020). From a clinical perspective, our study suggests that intervention in a social setting targeting transitions between adolescent and adulthood may be effective at preventing future relapse.

## Data Availability Statement

The raw data supporting the conclusions of this article will be made available by the authors, without undue reservation.

## Ethics Statement

The animal study was reviewed and approved by the University of Maryland College Park Animal Care and Use committee.

## Author Contributions

XL conceived the project, provided intellectual inputs, and wrote the manuscript. RA carried out experiments, analyzed the data, and wrote the manuscript. KG carried out experiments and analyzed the data. All authors contributed to the article and approved the submitted version.

## Conflict of Interest

The authors declare that the research was conducted in the absence of any commercial or financial relationships that could be construed as a potential conflict of interest.
