# Supplementary material for: Incubation of Oxycodone Craving Following Adult-Onset and Adolescent-Onset Oxycodone Self-Administration in Male Rats
Source: Front Behav Neurosci. 2021 Jun 23;15:697509. doi: 10.3389/fnbeh.2021.697509 (PMC8262493; doi:10.3389/fnbeh.2021.697509)
Supplement: Supplementary file 1 [file Table_1.docx]

5-7-21

Original research

**Incubation of oxycodone craving following adult-onset and adolescent-onset oxycodone self-administration in male rats**

Rachel D. Altshuler*, Kristine T. Garcia* and Xuan Li

**Table S1. Statistical analysis**

| **Figure Number** | **Test** | **F-value** | ***p*-value** | **Partial Eta^2^** |
| --- | --- | --- | --- | --- |
| Fig. 2A.  Training: oxycodone intake (single housing) | Infusion:  Training day (within)  Age (between)  Training day * age  Total intake (between) | F_9,405_=12.378  F_1,45_=0.443  F_9,405_= 1.442  t_45_= 0.666 | < 0.001*  0.509  0.168  0.509 | 0.216  0.010  0.031 |
| Fig. 2B.  Training: lever presses (single housing) | Training day (within)  Lever (within)  Age (between)  Training day * Age  Lever * Age  Training day * Lever  Training day * Lever * Age | F_9,405_= 6.330  F_1,45_= 114.018  F_1,45_= 0.169  F_9,405_= 1.704  F_1,45_= 4.053  F_9,405_= 8.866  F_9,405_= 0.733 | < 0.001*  < 0.001*  0.683  0.086  0.050  < 0.001*  0.678 | 0.123  0.717  0.004  0.036  0.083  0.165  0.016 |
| Fig. 2C  Incubation of oxycodone craving – single housing  Relapse tests: abstinence day 1 & 15 (30 min) | Lever (within)  Abstinence Day (within)  Age (between)  Lever * Age  Abstinence day * Age  Lever * Abstinence day  Lever * Abstinence day * Age  Post hoc (active lever):  Abstinence day (within): Adult  Adolescent  Age (between):  Abstinence day 1  Abstinence day 15  Time course (abstinence day 1):  Session Minute (within)  Age (between)  Session min * Age  Post hoc:  10 min  20 min  30 min  Time course (abstinence day 15):  Session minute (within)  Age (between)  Session min * Age | F_1,39_= 110.333  F_1,39_= 243.449  F_1,39_= 0.024  F_1,39_= 2.556  F_1,39_= 6.569  F_1,39_= 55.386  F_1,39_= 1.157  t_23_= -8.155  t_18_= --6.417  t_42_= 2.574  t_43_= -0.368    F_2,84_= 20.002  F_1,42_= 6.032  F_2,84_= 0.991  t_42_= 2.371  t_42_= 0.973  t_42_= 1.100  F_2,86_= 33.194  F_1,43_= 0.135  F_2,86_= 1.995 | < 0.001*  < 0.001*  0.879  0.118  0.014*  < 0.001*  0.289  <0.001*  <0.001*  0.014*  0.715  < 0.001*  0.018*  0.376  0.022*  0.336  0.277  < 0.001*  0.715  0.142 | 0.739  0.862  0.001  0.062  0.144  0.587  0.029  0.323  0.126  0.023  0.436  0.003  0.044 |
| Fig. 2D.  Incubation of oxycodone craving – single housing  Relapse tests: abstinence day 15 (60 min) | Lever (within)  Age (between)  Lever * Age  Time course (abstinence day 15):  Session min (within)  Age (between)  Session min * Age  Post hoc:  20 min  40 min  60 min | F_1,45_= 140.907  F_1,45_= 0.790  F_1,45_= 0.437  F_2,90_= 33.566  F_1,45_= 0.038  F_2,90_= 1.724  t_45_= -0.596  t_45_= 1.175  t_45_= -0.755 | < 0.001*  0.379  0.263  < 0.001*  0.847  0.184  0.554  0.246  0.454 | 0.758  0.017  0.010  0.427  0.001  0.037 |
| Fig. 3A  Training: oxycodone intake (group housing) | Infusion:  Training day (within)  Age (between)  Training day * Age  Total intake (between) | F_9,216_= 5.492  F_1,24_= 0.336  F_9,216_= 1.142  t_24_= 0.605 | 0.001*  0.551  0.334  0.551 | 0.186  0.015  0.045 |
| Fig. 3B  Training: oxycodone lever presses (group housing) | Training day (within)  Lever (within)  Age (between)  Training day * Age  Lever * Age  Training day * Lever  Training day * Lever * Age | F_9,216_= 3.228  F_1,24_= 59.884  F_1,24_= 0.494  F_9,216_= 0.656  F_1,24_= 2.231  F_9,216_= 6.652  F_9,216_= 1.155 | < 0.001*  0.001*  0.489  0.748  0.148  0.001*  0.325 | 0.119  0.714  0.020  0.027  0.085  0.217  0.046 |
| Fig. 3C  Incubation of oxycodone craving – group housing  Relapse tests: abstinence day 1 & 15 (30 min) | Lever (within)  Abstinence day (within)  Age (between)  Lever * Age  Abstinence day * Age  Lever * Abstinence day  Lever * Abstinence day * Age  Post hoc (active lever):  Abstinence day (within) Adult  Adolescent  Time course (abstinence day 1):  Session min (within)  Age (between)  Session min * Age  Post hoc:  10 min  20 min  30 min  Time course (abstinence day 15):  Session min (within)  Age (between)  Session min * Age | F_1,24_= 33.738  F_1,24_= 37.407  F_1,24_= 0.238  F_1,24_= 2.518  F_1,24_= 1.200  F_1,24_= 9.448  F_1,24_= 0.881  t_24_= -2.320  t_23_= -8.155  F_2,46_= 35.356  F_1,23_= 4.744  F_2,46_= 0.535  t_23_= 1.732  t_23_= 1.635  t_23_= 2.790  F_2,44_= 11.520  F_1,22_= 0.09  F_2,44_= 1.178 | < 0.001*  < 0.001*  0.630  0.126  0.284  0.005*  0.357  0.041*  < 0.001*  < 0.001*  0.040*  0.589  0.097  0.116  0.010*  < 0.001*  0.767  0.290 | 0.584  0.609  0.010  0.095  0.048  0.282  0.035    0.606  0.171  0.023  0.344  0.004  0.051 |
| Fig. 3D  Incubation of oxycodone craving – group housing  Relapse tests: Abstinence day 15 (60 min) | Lever (within)  Age (between)  Lever * Age  Time course (day 15):  Session min (within)  Age (between)  Session min * Age  Post hoc:  20 min  40 min  60 min | F_1,24_= 43.549  F_1,24_= 1.053  F_1,24_= 0.672  F_2,48_= 23.290  F_1,24_= 0.013  F_2,48_= 0.424  t_24_= -0.447  t_24_= 0.015  t_24_= 0.442 | < 0.001*  0.315  0.420  < 0.001*  0.911  0.657  0.659  0.988  0.662 | 0.645  0.042  0.027  0.492  0.001  0.017 |
| Fig. 4  Comparison of incubation slope across groups | Age (between)  Housing (between)  Age * Housing  Post hoc:  Age (between)  Single house  Group house | F_1,65_= 4.084  F_1,65_= 3.090  F_1,65_= 0.274  t_44_= -2.097  t_21_= -1.036 | 0.047*  0.083  0.602  p= 0.042*  p= 0.312 | 0.059  0.045  0.004 |
| Fig. 5A  Comparison of training between housing conditions | Adult:  Training day (within)  Housing (between)  Training day * Housing  Adolescent:  Training day (within)  Housing (between)  Training day * Housing | F_9,279_= 4.127  F_1,31_= 0.098  F_9,279_= 1.133  F_9,360_= 13.891  F_1,40_= 0.052  F_9,360_= 1.674 | < 0.001*  0.756  0.339  <0.001*  0.821  0.094 | 0.117  0.003  0.035  0.258  0.001  0.040 |
| Fig. 5B  Comparison of relapse tests between housing conditions | Abstinence day 1 (30 min):  Age (between)  Housing (between)  Age * Housing  Abstinence day 15 (60 min):  Age (between)  Housing (between)  Age * Housing  Post hoc [Housing(between)]:  Adult  Adolescent | F_1,65_= 11.439  F_1,65_= 1.500  F_1,65_= 0.243  F_1,67_= 0.080  F_1,67_= 8.243  F_1,67_= 0.001  t_45_= 1.745  t_37_= 2.381 | 0.001*  0.225  0.624  0.779  0.005*  0.970  0.090  0.023* | 0.150  0.023  0.004  0.001  0.110  0.000 |
